# Supplementary material for: Comparison of Immune Responses between Inactivated and mRNA SARS-CoV-2 Vaccines Used for a Booster Dose in Mice
Source: Viruses. 2023 Jun 11;15(6):1351. doi: 10.3390/v15061351 (PMC10301383; doi:10.3390/v15061351)
Supplement: Supplementary file 1 [file viruses-15-01351-s001.zip › viruses-2426911-supplementary.pdf]

Supplemental Figures and Figure legends

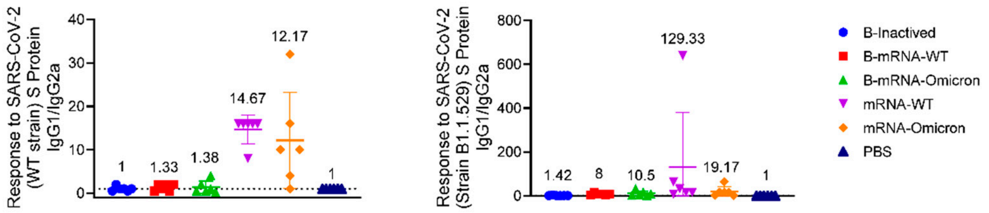

Figure S1. Antibody titers detected by enzyme-linked immunosorbent assay (ELISA). S protein of strain WT (left) and strain B1.1.529 (right) were precoated in the microplates, the responses of IgG1 (Figure 2B& 2E in the main text) and IgG2a (Figure 2C& 2F in the main text) with sera from different groups were detected. The ratio of IgG1/IgG2a were calculated from the IgG1 and IgG2a titers of each mouse, dash line means the IgG1/IgG2a=1, mean values of all groups were shown in the top of each bar. N=6, points represent individual mice.
